# Laparoscopic anterior gastropexy for type III/IV hiatal hernia in elderly patients

**DOI:** 10.1186/s40792-017-0323-1

**Published:** 2017-03-20

**Authors:** Shigeyoshi Higashi, Kiyokazu Nakajima, Koji Tanaka, Yasuhiro Miyazaki, Tomoki Makino, Tsuyoshi Takahashi, Yukinori Kurokawa, Makoto Yamasaki, Shuji Takiguchi, Masaki Mori, Yuichiro Doki

**Affiliations:** 1 Department of Gastroenterological Surgery, Osaka University Graduate School of Medicine, Osaka, Japan; 2 Division of Next Generation Endoscopic Intervention (Project ENGINE), Global Center for Advanced Medical Engineering and Informatics, Osaka University, Suite 0912, Center of Medical Innovation and Translational Research 2-2, Yamadaoka, Suita, Osaka 565-0871 Japan

**Keywords:** Large esophageal hiatal hernia, Anterior gastropexy, Elderly patients

## Abstract

**Introduction:**

Large esophageal hiatal hernias occur most commonly in elderly patients with comorbidities, in whom even an elective surgery cannot be performed without high risks. Although fundoplication is recommended for esophageal hiatal hernia repair, we prefer not to limit our options to fundoplication, as obstruction is a frequent main complaint. We favor an anterior gastropexy approach instead to perform anti-reflux surgery and prevent recurrent protrusion and torsion of the incarcerated organ with minimal risk. The aim was to evaluate the safety and effectiveness of anterior gastropexy for large hiatal hernia in elderly patients with comorbidities.

**Case presentation:**

We retrospectively evaluated 8 patients who underwent laparoscopic anterior gastropexy for large hiatal hernia (type III or IV) since 2006. All patients were women with a median age of 82 years (range, 74–87 years). The major complaint was obstruction in all patients, with relatively mild reflux symptoms. They underwent successful laparoscopic surgery with no conversion to laparotomy.

Fundoplication was performed in 4 cases. No perioperative complications occurred, and the main complaint resumed rapidly in all patients, without recurrence during postoperative follow-up of median 48 months (range, 5–77 months).

**Conclusion:**

Laparoscopic anterior gastropexy is safe and effective and can be considered as one of the practical surgical options for large hiatal hernias in elderly patients, whom surgical intervention should be minimized due to their comorbidities.

## Background

The surgical treatment is recommended for patients with large esophageal hiatal hernia. However, as large hiatal hernias most commonly occur in elderly female patients with comorbidities and lumbar kyphosis in Japan, even an elective surgery cannot be performed without high risks [1–3]. In light of the fact that the major complaint in this population of patients is passage disturbance due to anatomical distortion, we consider that the treatment focus should be placed on the repair of the esophageal hiatus, the prevention of recurrent herniation, and the prevention of the volvulus of incarcerated organs. Although most surgeons prefer addition of anti-reflux surgery (i.e., fundoplication) during esophageal hiatal hernia repair to prevent reflux symptoms, this technique may increase the risk of dysphagia [4]. However, due to deformity of the stomach associated with long-term herniation, performing “floppy” fundoplication during hernia repair is often technically difficult in those patients and may induce postoperative obstruction and bloating. Thus, the suitability of fundoplication remains controversial [5–7].

Since 2006, we have selectively performed fixation of the anterior wall of the stomach (anterior gastropexy) in addition to esophageal hiatal hernia repair in elderly patients with type III/IV hiatal hernia. The aim of this study was to evaluate the safety and effectiveness of anterior gastropexy for large hiatal hernia in this population of patients.

## Case presentation

### Patient characteristics

**Fig. 1:**
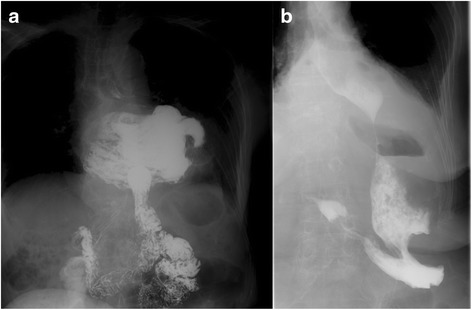
The type III/IV hiatal hernia before and after surgery. Upper gastrointestinal series is a large hiatus hernia (type III or IV) patient at preoperative (**a**) and postoperative (**b**)

Laparoscopic surgery was attempted in 8 patients with large hiatal hernia (type III or IV) (Fig. 1a) since 2006. The patient characteristics are summarized in Table 1. All patients were women with a median age of 82 years (range, 74–87 years) and a body mass index of 24.7 kg/m^2^ (14.6–29.8 kg/m^2^). Lumbar kyphosis was observed in 6 cases. Their major preoperative complaint was obstruction, with mild reflux symptoms in all patients. Their comorbidities were shown in Table 1: hypertension (4 cases), coronary artery disease (2), rheumatoid arthritis (1), abdominal aortic aneurysm (1), and amyotrophic lateral sclerosis (with duplicates) (1). The median duration of the symptoms was 12 months (range, 6–36 months).

**Table 1:** Patient characteristics

Age, year (range)	82 (74–86)
Sex, male/female	0/8
Hernia type (III/IV)	5/3
BMI, kg/m^2^ (range)	24.7 (14.6–25.8)
Lumbar kyphosis, *n* (%)	6 (75%)
Comorbidities, *n* (%)	
Hypertension	4 (50)^a^
Coronary artery disease	2 (25)^a^
Rheumatoid arthritis	1 (12.5)
Abdominal aortic aneurysm	1 (12.5)^a^
Amyotrophic lateral sclerosis	1 (12.5)
Symptoms, *n* (%)	
Obstruction	8 (100)
Vomiting	2 (25)
Chest pain	2 (25)
Heartburn	1 (12.5)
Morbidity period, month (range)	12 (6–36)

^a^Duplicate complication

### Surgical technique

**Fig. 2:**
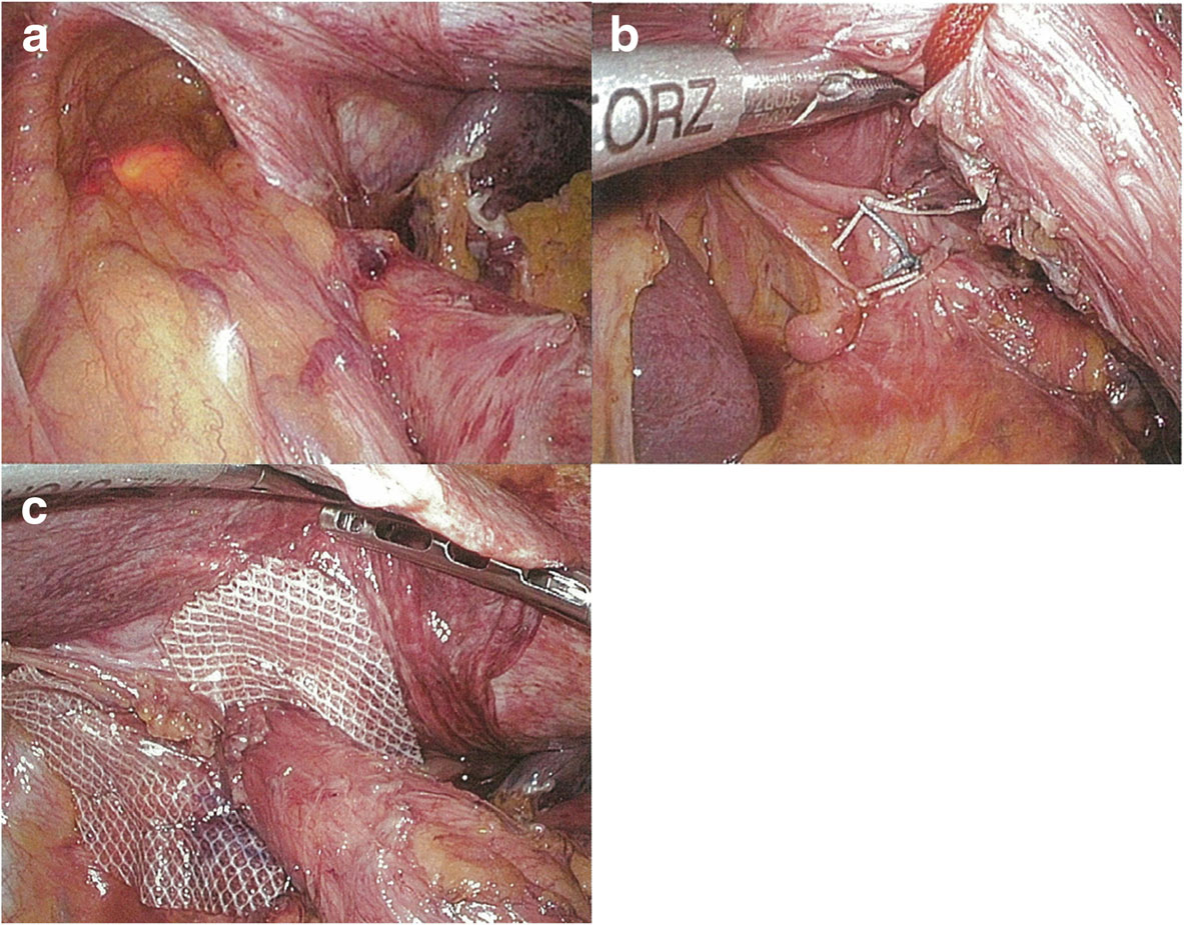
Hiatus repair. The hiatus (**a**) on the dorsal side of the esophagus was closed (**b**), and additional mesh reinforcement was provided (**c**)

**Fig. 3:**
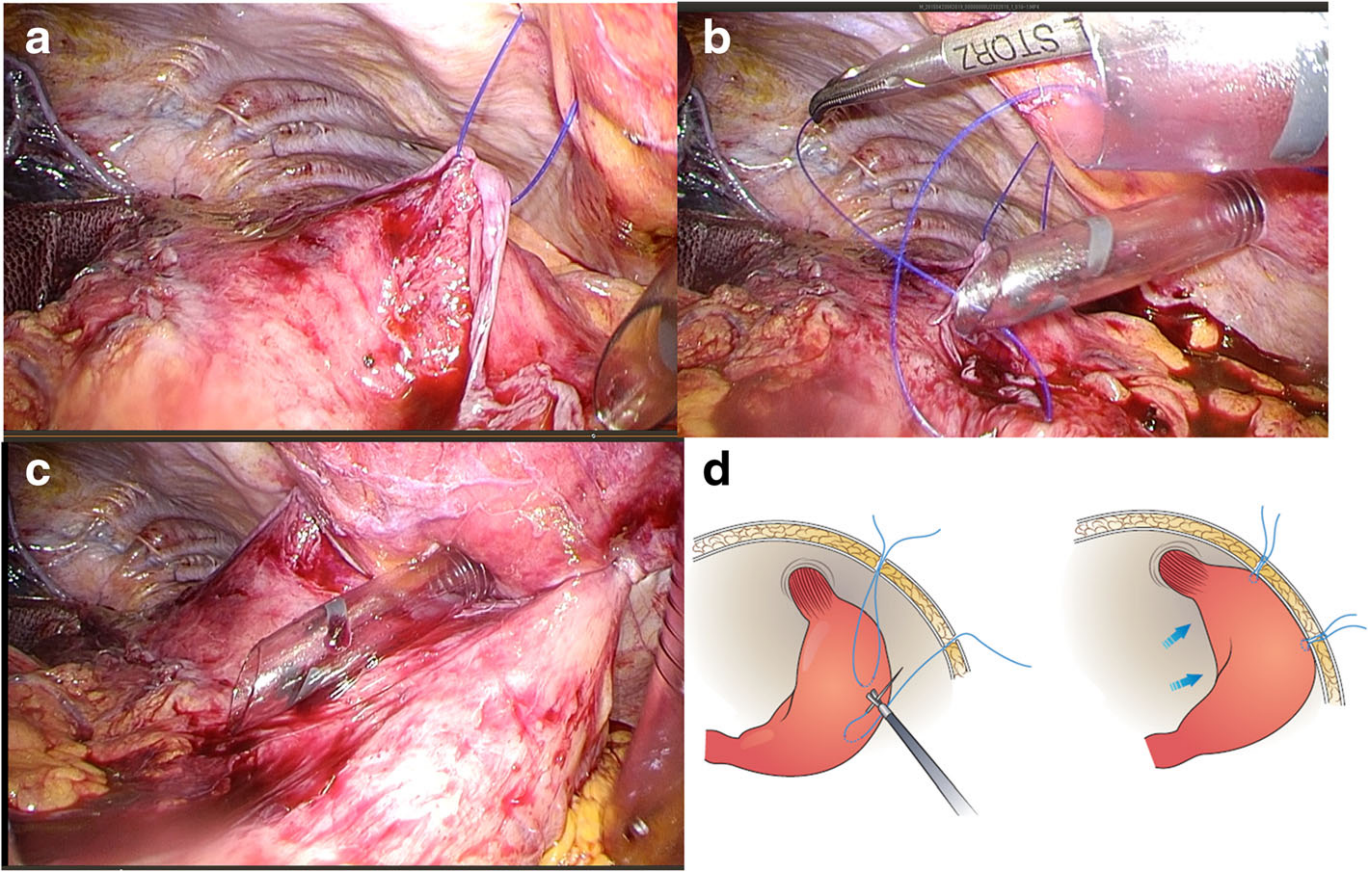
Anterior gastropexy. Suturing the anterior stomach wall to the anterior abdominal wall at 2 sites using 2-0 Prolene (**a**, **b**, **d**) fixing the gastric wall on the anterior layer of the rectus sheath (subcutaneous) (**c**, **d**)

In all cases, conventional 5-port technique is used. The hernia sac that protrudes into the mediastinum is resected, and the abdominal esophagus and gastric cardia are reduced into the peritoneal cavity. The hiatus (Fig. 2a) on the dorsal side of the esophagus is closed with non-absorbable sutures using the interrupted technique (Fig. 2b), and additional mesh reinforcement is provided when indicated (Fig. 2c). If the tissue around the hiatus is intact, the hiatal repair is performed using direct closure technique. If there is no deformity in the stomach, standard floppy Nissen fundoplication utilizing the fundus of the stomach is performed under the calibration of the gastroesophageal junction with the flexible endoscope (GIF-XQ260 Olympus Medical System, Tokyo, Japan). The diameter of the endoscope is 9.0 mm (27 Fr). We do not use standard esophageal bougie with 44 Fr, since elderly Japanese patients do not allow passage of this size of bougie. We form a 2.0-cm fundic wrap with 2 or 3 sutures. We do not place any sutures on the posterior gastric wall to create the wrap; instead, we place sutures on the anterior wall according to Rossetti’s original fundoplication method [8]. This is crucial to fashion “floppy” fundoplication, followed by the anterior gastropexy. Two fixation points are determined at locations slightly closer to the greater curvature on the anterior wall of the gastric corpus. A 2-0 Prolene suture on a straight needle is passed through the abdominal wall to capture the target gastric wall within the peritoneal cavity and passed through the abdominal wall again to ligate and fix the gastric wall on the anterior layer of the rectus sheath subcutaneously (Fig. 3a–d). The suture is then ligated on the fascia after confirming the status of stomach fixation by intraoperative endoscopy. Conclusion laparoscopy and endoscopy are performed to confirm the absence of axial deformity, stricture, or damage of the esophagus.

### Perioperative data

All cases underwent successful laparoscopic surgery, without any conversion to laparotomy. The hiatus was repaired in all cases with mesh or direct suture. Six out of 8 cases underwent hiatal mesh repair, and 4 (50%) cases underwent short and floppy Nissen fundoplication utilizing the fundus of the stomach. Anterior gastropexy with or without fundoplication was examined. There was no significant difference in surgical time (267 vs. 231 min *p* = 0.54), bleed loss (23 vs. 30 ml *p* = 0.37), and postoperative length of the hospital stay (18 vs. 16 day *p* = 0.66) with or without fundoplication. Patients experienced a rapid and uneventful postoperative recovery, and none had perioperative complications (Table 2).

**Table 2:** Perioperative data

	Anterior gastropexy with fundoplication (*n* = 4)	Anterior gastropexy without fundoplication (*n* = 4)
Operative time, min (range)	267 (203–289)	231 (223–271)
Blood loss, ml (range)	23 (5–30)	30 (10–190)
Laparoscopic surgery, *n* (%)	4 (100)	4 (100)
Hiatal repair		
With mesh, *n* (%)	2 (50)	4 (100)
Without mesh, *n* (%)	2 (50)	0 (0)
Perioperative complications	0 (0%)	0 (0%)
Postoperative length of stay, day (range)	18 (12–25)	16 (13–27)

### Follow-up

**Fig. 4:**
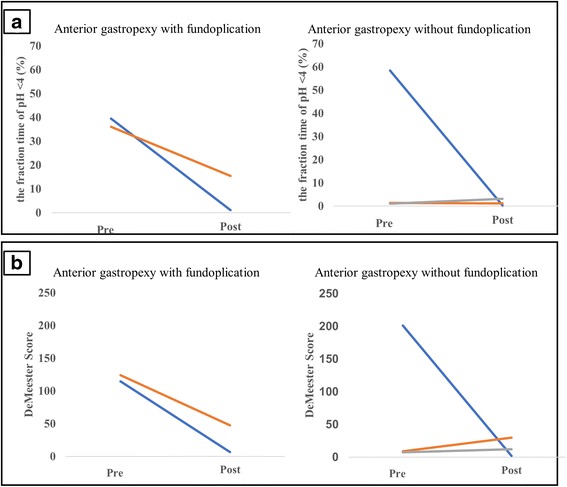
Twenty-four-hour pH monitoring. The fraction time of pH <4 (%) improved (*n* = 4) or maintained normal (*n* = 1) in all 5 patients (**a**). The DeMeester score improved (*n* = 3) or maintained normal (*n* = 1) (**b**). The remaining 1 patient showed deterioration of DeMeester score (preoperative score 8.8 vs. postoperative score 29.7)

Upper gastrointestinal endoscopy and barium swallow were performed in 1 year after surgery, revealing no esophageal hiatal hernia recurrence. In addition, 24-h pH monitoring revealed that the fraction time of pH <4 (%) improved (*n* = 3) or maintained normal (*n* = 2) in all 5 patients (Fig. 4a). The DeMeester score improved (*n* = 3) or maintained normal (*n* = 1) (Fig. 4b). The remaining 1 patient showed deterioration of DeMeester score (preoperative score 8.8 vs. postoperative score 29.7). Although mild obstruction remains in 2 patients who underwent anterior gastropexy with fundoplication, overall symptoms were improved in all patients. One patient who underwent anterior gastropexy without fundoplication complained of mild postoperative reflux symptoms. Although this patient continued taking oral proton pump inhibitors, the reflux was clinically mild and reflux esophagitis was endoscopically assessed as grade A using the Los Angeles classification, without serious comorbidities such as aspiration pneumonia, suggesting good reflux control (Table 3).

**Table 3:** Postoperative outcome

	Anterior gastropexy with fundoplication (*n* = 4)	Anterior gastropexy without fundoplication (*n* = 4)
Hernia recurrence, *n* (%)	0 (0)	0 (0)
Residual mild obstruction, *n* (%)	2 (50)	0 (0)
Medication (proton pump inhibitor), *n* (%)	0 (0)	1 (25)
Endoscopically proven esophagitis, *n* (%)	0 (0)	1 (25)

### Discussion

Esophageal hiatal hernia is increasing in Japan due to the westernization of lifestyle and subsequent increasing obesity. In Japan, advanced age, rather than obesity, is still the predominant risk factor for developing type III/IV hiatal hernia [9]. As most large esophageal hiatal hernia occurs commonly in elderly patients with medical/surgical comorbidities, surgical intervention cannot be performed without additional risks, with an operative mortality rate of 1.38% even for elective surgeries [1].

The first issue is the selection of the operative technique for large esophageal hiatal hernia still remains controversial, as 40% of patients with type III hernia are reported to experience asymptomatic recurrence most often within 1 year after hernia repair alone [10–12]. The choice of primary hiatal closure or mesh repair is also a topic of debate [13]. Although the proportion of patients with recurrence was 9% in the mesh group, those in the primary group had a recurrence rate of 22% 6 months after surgery [14]. However, patients with mesh and without mesh had similar recurrence rate after approximately 5 years postoperatively [15]. Interpretation of data might change at the different time point during the observation period.

The second issue is the addition of concomitant anti-reflux surgery (i.e., fundoplication) at the time of hernia repair, to prevent progression of postoperative reflux symptoms. However, the addition of fundoplication may often increase the rate of swallowing disorders. In patients undergoing surgery for a large hiatal hernia with a symptom of obstruction, mobilization around the esophageal hiatus may partially damage physiological anti-reflux function of the gastroesophageal junction, resulting in reflux symptoms. In addition, fundoplication is often technically difficult in these patients due to deformity of the stomach associated with long-term herniation and/or axial volvulus. On the other hand, approximately 65% of patients without concomitant fundoplication at hiatal hernia repair reported experiencing reflux symptoms [16, 17]. Since our patients commonly complained of obstructive symptoms preoperatively, the authors have decided not to perform fundoplication as routine. The addition of fundoplication for those patients, which we no longer consider, might lead to failure of symptom relief. First, we try to place the fully mobilized fornix inside the retro-esophageal space to confirm its mobility when the fornix stays in situ without spontaneous rotation; we consider addition of traditional Nissen fundoplication. When the fornix does not stay in the space due to deformity of the stomach, we do not perform Nissen fundoplication. Instead, we have actively performed anterior gastropexy. To our knowledge, this study is the first surgical report that describes technical details and outcomes in Japan. Our patients had advanced age (median 82 years old) and high incidence of lumbar kyphosis (75%). All had medical comorbidities with various degrees. Surgery was carefully indicated, and surgical technique was flexibly selected intra-operatively. In such group of patients, the immediate and mid-term postoperative follow-up examinations showed generally favorable surgical outcomes without major complications. There was no serious obstruction in 4 cases with fundoplication and in 1 patient without fundoplication. Although there was mild reflux symptom in 1 patient, the anterior gastropexy was considered safe and practical with acceptable surgical outcomes. The short and floppy fundoplication was only possible in 4 out of 8 patients, but the relationship between reflux symptoms in patients with/without fundoplication was not clear. The authors believe that addition of fundoplication should not be a routine in this population and indicated only for selected cases with less gastric deformity and tissue weakness.

One major limitation in our study is its small number of patients. Further clinical studies with larger number of patients, ideally in a multi-center setting, are definitely required.

## Conclusions

Laparoscopic anterior gastropexy is safe and effective and can be considered as one of the attractive surgical alternatives for type III/IV hiatal hernias in elderly patients, whom surgical intervention should be minimized due to their comorbidities and preoperative symptoms.
